# Information Geometry for Radar Target Detection with Total Jensen–Bregman Divergence

**DOI:** 10.3390/e20040256

**Published:** 2018-04-06

**Authors:** Xiaoqiang Hua, Haiyan Fan, Yongqiang Cheng, Hongqiang Wang, Yuliang Qin

**Affiliations:** 1 School of Electronic Science, National University of Defence Technology, Changsha 410073, China; 2 Space Engineering University, Beijing 101400, China

**Keywords:** information geometry, Hemitian positive-definite matrix, total Jensen–Bregman divergence, median matrix, radar target detection

## Abstract

This paper proposes a radar target detection algorithm based on information geometry. In particular, the correlation of sample data is modeled as a Hermitian positive-definite (HPD) matrix. Moreover, a class of total Jensen–Bregman divergences, including the total Jensen square loss, the total Jensen log-determinant divergence, and the total Jensen von Neumann divergence, are proposed to be used as the distance-like function on the space of HPD matrices. On basis of these divergences, definitions of their corresponding median matrices are given. Finally, a decision rule of target detection is made by comparing the total Jensen-Bregman divergence between the median of reference cells and the matrix of cell under test with a given threshold. The performance analysis on both simulated and real radar data confirm the superiority of the proposed detection method over its conventional counterparts and existing ones.

## 1. Introduction

**Figure 1:**
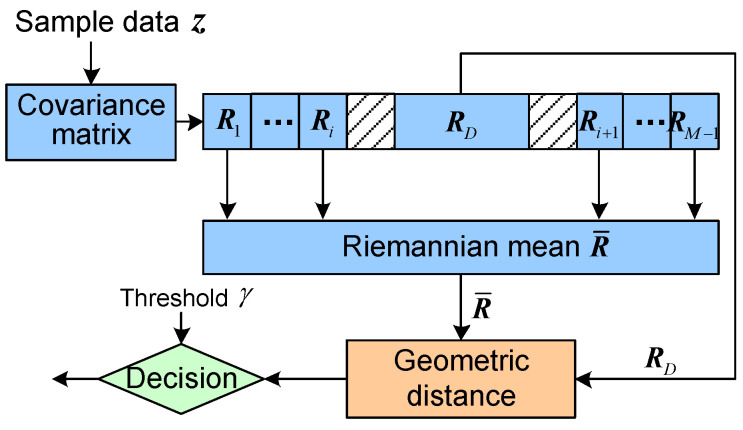
Riemannian mean-based geometric detector.

It is important to improve the performance of target detection in a clutter for a radar system. Due to the limitation of sample data, classical constant false alarm rate (CFAR) detector based on fast Fourier transform (FFT) [1] experiences performance degradation. This is because, the energy of the Doppler filter banks spreads and the Doppler resolution is poor, when the correlation of the sample data is captured by the FFT. To address these problems, Barbaresco investigated a geometric detection algorithm on a Hermitian positive-definite (HPD) matrix manifold, named as Riemannian distance-based geometric detector [2]. An HPD matrix $R_{i}$ is utilized to model the information of each cell, as the sample data z is assumed to be following a complex circular Gaussian distribution with zero mean. Then, the detection statistic in each cell is the Riemannian distance of the matrix $R_{D}$ of the cell under test and the mean $\bar{R}$ of its surrounding cells. Finally, the detection statistic is compared with a threshold γ to determine the decision about “target absence” or “target presence”. It can be referred to Figure 1.

In this geometric detector, the sample data of each cell in one coherent processing interval (CPI) is represented by an HPD matrix. According to this parameterization, the detection method is performed on the HPD matrix manifold, and the geometric characteristic is considered. Then, the metric is derived [2,3], and the existence and uniqueness of geometric mean had been proven in [4]. The geometric detector has been used to monitor the turbulence of a plane [5,6,7], target detection in coastal X-band and HF surface wave radars [2,3]. Experiment results show that this geometric detector outperforms the classical FFT-CFAR detector [2].

The classical FFT-CFAR detector has a similar scheme to the geometric detector. The main differences between them lie in the following three aspects: (1) the correlation of sample data is captured by an HPD matrix, and not the FFT coefficient; (2) the Riemannian distance is utilized in the detector, and not the Euclidean distance; and (3) the average value of HPD matrices is the geometric mean, rather than the arithmetic average. These differences illuminate that different geometry space is considered in these two detectors.

Many divergences have been used as a distance-like function on the space of HPD matrices. For instance, the Hellinger distance has been utilized to design a geometric target detector in a clutter; the Bhattacharyya divergence is used for filtering in medical imaging [8,9]; and the Log-Euclidean distance has been employed to measure the dissimilarity of two HPD matrices [10]. In these contexts, geometric measures have achieved performance improvement. In [11], the total Bregman divergence is defined on the convex function space, and many perfect properties have been analyzed in theory. The t-center of HPD matrices have been derived according to the definition of total Bregman divergence. It has been applied to diffusion tensor magnetic resonance image analysis [11], object tracking [12], and shape retrieval [13,14]. More recently, the definition of total Jensen–Bregman divergence has been presented on the space of convex functions in [15,16]. This class of divergence has not been applied in practice. In [17], a Kullback–Leibler divergence-based geometric target detection algorithm is proposed, and experimental results give a proof of performance improvement when compared to the classical FFT-CFAR detector. In [18,19], we have analyzed the anisotropy of the metric used in our geometric detection method. In particular, we have found that the detection performance of geometric detector is related to the measure used. In [20], the definition of total Bregman divergence has been extended on the Riemannian manifold, and we have validated the superiority of performance of the total Bregman divergence-based geometric detector when compared with the Riemannian distance-based geometric detector. These results inspire us to study new measures.

In this work, the definition of total Jensen–Bregman divergence is presented on the HPD matrix manifold. Specially, the total Jensen square loss, the total Jensen von Neumann divergence, and the total Jensen log-determinant divergence are proposed and defined. Based on three divergences, medians of HPD matrices are presented. As a result, a total Jensen–Bregman divergence-based radar target detection is developed. The main contribution of this paper is that a new class of total Jensen–Bregman divergence is proposed for designing the target detector on the Riemannian manifold.

The rest of this paper is organized as follows. In Section 2, we give a description about the signal model and signal manifold. In Section 3, the basic mathematical knowledge of matrix information geometry is presented. In particular, the definition of total Jensen–Bregman divergence is given. The median matrix associated with total Jensen–Bregman divergence is derived in Section 4. Then, in Section 5, we evaluate performances of total Jensen–Bregman divergences-based target detection algorithms as well as the Riemannian distance-based detector and the FFT-CFAR detector by simulated and real sea clutter data. Finally, a conclusion is provided in Section 6.

### Notation

Here are some notations for the descriptions of this article. A scalar *x* is denoted using the math italic. A matrix A and a vector x are noted as uppercase bold and lowercase bold, respectively. The conjugate transpose of matrix A is denoted as $A^{H}$. tr(A) is the trace of matrix A. det(A) is the determinant of matrix A. I is the identity matrix. All *n*-dimensional vectors are noted by C(n). H(n) is the set of all n×n Hermitian matrices. ${∥A∥}_{F}$ denotes the F-norm of A. All n×n positive-definite matrices in H(n) consist of the space P(n). Finally, E(·) denotes the statistical expectation.

## 2. Signal Model and Signal Manifold

The radar usually receives the echo returned from a moving target, and the phase information is included in the echo data. The correlation of the sample data $z=\{z_{1},z_{2},\cdots ,z_{n}\}$ can be used to capture the Doppler information of the target. Here, the data is assumed to follow a complex multivariate Gaussian distribution with zero mean, z∼CN(0,R) [2],

(1) $$\begin{array}{c} p\left(z\left|R\right.\right)=\frac{1}{\pi^{n}\det\left(R\right)}\exp\left\{-z^{H}R^{-1}z\right\} \end{array}$$

where the matrix R is given as [2],

(2) $$\begin{array}{c} R=\left[zz^{H}\right]=\left[\begin{array}{c} \begin{array}{cccc} r_{0} & {\bar{r}}_{1} & \cdots & {\bar{r}}_{n-1} \end{array}\\ \begin{array}{cccc} r_{1} & r_{0} & \cdots & {\bar{r}}_{n-2} \end{array}\\ \begin{array}{cccc} \vdots & \ddots & \ddots & \vdots \end{array}\\ \begin{array}{cccc} r_{n-1} & \cdots & r_{1} & r_{0} \end{array} \end{array}\right]\\ r_{k}=\left[z_{i}{\bar{z}}_{i+k}\right],0\leq k\leq n-1,0\leq i\leq n-1 \end{array}$$

where ${\bar{r}}_{i}$ denotes the conjugate of $r_{i}$, and $r_{k}$ is the coefficient of the sample data. Due to the limitation of number of the sample data, according to the ergodicity, the statistical expectation $E[z_{i}{\bar{z}}_{i+k}]$ can be computed by a finite time serials,

(3) $$\begin{array}{c} {\hat{r}}_{k}=\frac{1}{n-k}\sum_{j=0}^{n-1-k}z_{j}{\bar{z}}_{j+k},0\leq k\leq n-1 \end{array}$$

The sample data $z=\{z_{1},z_{2},\cdots ,z_{n}\}$ of each cell in one CPI is represented by a Toeplitz matrix R. In particular, the sample data z lies in the linear space, while the matrix R is considered on the matrix manifold. Thus, the transformation can be given as,

(4) $$\begin{array}{c} \Psi :C(n)\to P(n),z\to R\in (n) \end{array}$$

P(n) constitutes a differentiable non-linear manifold [21] with non-positive curvature [22,23]. Then, the target detection is performed on the Riemannian manifold. The manifold P(n) is a symmetric space [24], and more details are referred to [25].

## 3. Matrix Information Geometry

In this Section, we will introduce some basic mathematical knowledge of matrix information geometry. For instance, the geometry of Riemannian manifold of HPD matrices, and distance measures, are presented. Moreover, the definition of total Jensen–Bregman divergence is presented.

### 3.1. The Geometry of Riemannian Manifold

For a n×n matrix A, A is a Hermitian matrix if $A^{H}=A$. H(n) denotes the linear space constituted of n×n Hermitian matrices. A is noted as a positive semi-definite if $x^{H}Ax\geq 0,\forall x\in C\left(n\right)$. In addition, if A is invertible, then, we can say A is a positive-definite Hermitian matrix. All these HPD matrices are consisting of a convex symmetric cone [26],

(5) $$\begin{array}{c} P\left(n\right)=\left\{A\in H\left(n\right),A>0\right\} \end{array}$$

$P\left(n\right)$ forms a non-linear manifold, and an HPD matrix is a point on the manifold. For any point A, its tangent space $T_{A}$ is the space $H\left(n\right)$. In particular, the infinitesimal arclength at point A is given as [26],

(6) $$\begin{array}{c} ds:={\left(tr{\left(A^{-1}dA\right)}^{2}\right)}^{1/2\;}={∥A^{-1/2\;}dAA^{-1/2\;}∥}_{F} \end{array}$$

The metric is defined by the infinitesimal on P(n). For any a point A, the inner product can be defined as,

(7) $$\begin{array}{c} {\langle R_{1},R_{2}\rangle }_{A}=tr\left(A^{-1}R_{1}A^{-1}R_{2}\right),{∥R_{1}∥}_{A}={\langle R_{1},R_{1}\rangle }_{A}^{1/2\;} \end{array}$$

The distance between two points $R_{1}$, $R_{2}$ on the manifold is given by [26],

(8) $$\begin{array}{c} d_{R}^{2}\left(R_{1},R_{2}\right)={∥\mathrm{logm}\left(R_{1}^{-1/2\;}R_{2}R_{1}^{-1/2\;}\right)∥}_{F}^{2}=\sum_{k=1}^{n}\log^{2}\left(\lambda_{k}\right) \end{array}$$

where $\lambda_{k}$ is the $k^{th}$ eigenvalue of $R_{1}^{-1/2\;}R_{2}R_{1}^{-1/2\;}$, and logm(·) is a logarithmic map.

### 3.2. Total Jensen–Bregman Divergence on the Riemannian Manifold

The definition of total Jensen–Bregman divergence is given by Frank Nielsen in [16] on the space of convex functions, and many properties have been analyzed from the perspective of algebra and geometry. Given a strictly convex and differentiable function *f*, for x,y∈R, the total Jensen–Bregman divergence δ between *x*, *y* is defined as,

(9) $$\delta_{\alpha }\left(x,y\right)=\frac{1}{\alpha \left(1-\alpha \right)}\rho \left(x,y\right)\left({\left(f\left(x\right)f\left(y\right)\right)}_{\alpha }-f\left({\left(xy\right)}_{\alpha }\right)\right)\;\rho \left(x,y\right)=1/\sqrt{1+\frac{\langle f\left(x\right)-f\left(y\right),f\left(x\right)-f\left(y\right)\rangle }{\langle x-y,x-y\rangle }}\;,\;\alpha \in \left[0,1\right]$$

where $f\left({\left(xy\right)}_{\alpha }\right)=\alpha f\left(x\right)+\left(1-\alpha \right)f\left(y\right)$ and ${\left(xy\right)}_{\alpha }=\alpha x+\left(1-\alpha \right)y$. α is a skew scale parameter. In the following, the definition of total Jensen–Bregman divergence is extended from the space of convex functions to Riemannian manifold of HPD matrices. Furthermore, according to different forms of the function *f*, we define three new divergences, namely the total Jensen square loss, the total Jensen von Neumann divergence, and the total Jensen Log-determinant divergence.

**Definition** **1.** *Let a strictly convex function f be differentiable, the total Jensen–Bregman divergence between two HPD matrices X, Y is defined as,*

(10) $$\begin{array}{ccc} \delta_{\alpha }\left(X,Y\right) & = & \frac{1}{\alpha \left(1-\alpha \right)}\rho \left(X,Y\right)tr\left({\left(f\left(X\right)f\left(Y\right)\right)}_{\alpha }-f\left({\left(\mathrm{XY}\right)}_{\alpha }\right)\right)\\ \rho \left(X,Y\right) & = & 1/\sqrt{1+\frac{{∥f\left(X\right)-f\left(Y\right)∥}_{F}^{2}}{{∥X-Y∥}_{F}^{2}}}\;,\;\alpha \in \left[0,1\right] \end{array}$$

In the following, we define three divergences when the function *f* has various forms.

If $f\left(x\right)=x^{2}$, then $tr\left(f\left(X\right)\right)=tr\left(X^{2}\right)$, and (10) can be rewritten as,

(11) $$\begin{array}{c} \delta_{\alpha }\left(X,Y\right)\\ =\frac{1}{\alpha \left(1-\alpha \right)}\rho \left(X,Y\right)tr\left({\left(X^{2}Y^{2}\right)}_{\alpha }-{\left({\left(\mathrm{XY}\right)}_{\alpha }\right)}^{2}\right)\\ =\frac{1}{\alpha \left(1-\alpha \right)}\rho \left(X,Y\right)tr\left(\alpha X^{2}+\left(1-\alpha \right)Y^{2}-{\left(\alpha X+\left(1-\alpha \right)Y\right)}^{2}\right)\\ \rho \left(X,Y\right)=1/\sqrt{1+\frac{{∥X^{2}-Y^{2}∥}_{F}^{2}}{{∥X-Y∥}_{F}^{2}}}\;,\;\alpha \in \left[0,1\right] \end{array}$$

Equation (11) denotes the total Jensen square loss.

Let $f\left(x\right)=x\log x-x$, then $tr\left(f\left(X\right)\right)=tr\left(X\log\left(X\right)-X\right)$, and (10) yields the divergence,

(12) $$\begin{array}{c} \delta_{\alpha }\left(X,Y\right)=\frac{1}{\alpha \left(1-\alpha \right)}\rho \left(X,Y\right)\\ \times tr\left\{{\left(\left(X\log\left(X\right)-X\right)\left(Y\log\left(Y\right)-Y\right)\right)}_{\alpha }-\left(Z\log\left(Z\right)-Z\right)\right\}\\ =\frac{1}{\alpha \left(1-\alpha \right)}\rho \left(X,Y\right)\\ \times tr\left(\alpha \left(X\log\left(X\right)-X\right)+\left(1-\alpha \right)\left(Y\log\left(Y\right)-Y\right)-\left(Z\log\left(Z\right)-Z\right)\right)\\ \rho \left(X,Y\right)=1/\sqrt{1+\frac{{∥\left(X\log\left(X\right)-X\right)-\left(Y\log\left(Y\right)-Y\right)∥}_{F}^{2}}{{∥X-Y∥}_{F}^{2}}}\;\\ Z=\alpha X+\left(1-\alpha \right)Y,\alpha \in \left[0,1\right] \end{array}$$

We call Equation (12) the total Jensen von Neumann divergence.

If $f\left(x\right)=-\log x$, then $tr\left(f\left(X\right)\right)=-\log\left(\det\left(X\right)\right)$, and (10) can be rewritten as,

(13) $$\begin{array}{c} \delta_{\alpha }\left(X,Y\right)\\ =\frac{1}{\alpha \left(1-\alpha \right)}\rho \left(X,Y\right)tr\left({\left(\left(-\log\left(X\right)\right)\left(-\log\left(Y\right)\right)\right)}_{\alpha }+\log\left({\left(\mathrm{XY}\right)}_{\alpha }\right)\right)\\ =\frac{1}{\alpha \left(1-\alpha \right)}\rho \left(X,Y\right)\\ \times tr\left(\log\left(\alpha X+\left(1-\alpha \right)Y\right)-\alpha \log\left(X\right)-\left(1-\alpha \right)\log\left(Y\right)\right)\\ =\frac{1}{\alpha \left(1-\alpha \right)}\rho \left(X,Y\right)\\ \times \left(\log\det\left(\alpha X+\left(1-\alpha \right)Y\right)-\alpha \log\det\left(X\right)-\left(1-\alpha \right)\log\det\left(Y\right)\right)\\ \rho \left(X,Y\right)=1/\sqrt{1+\frac{{∥\log\left(X\right)-\log\left(Y\right)∥}_{F}^{2}}{{∥X-Y∥}_{F}^{2}}}\;,\alpha \in \left[0,1\right] \end{array}$$

Equation (13) is named as the total Jensen Log-determinant divergence, or total Jensen Stein loss.

### 3.3. Anisotropy Analysis

**Figure 2:**
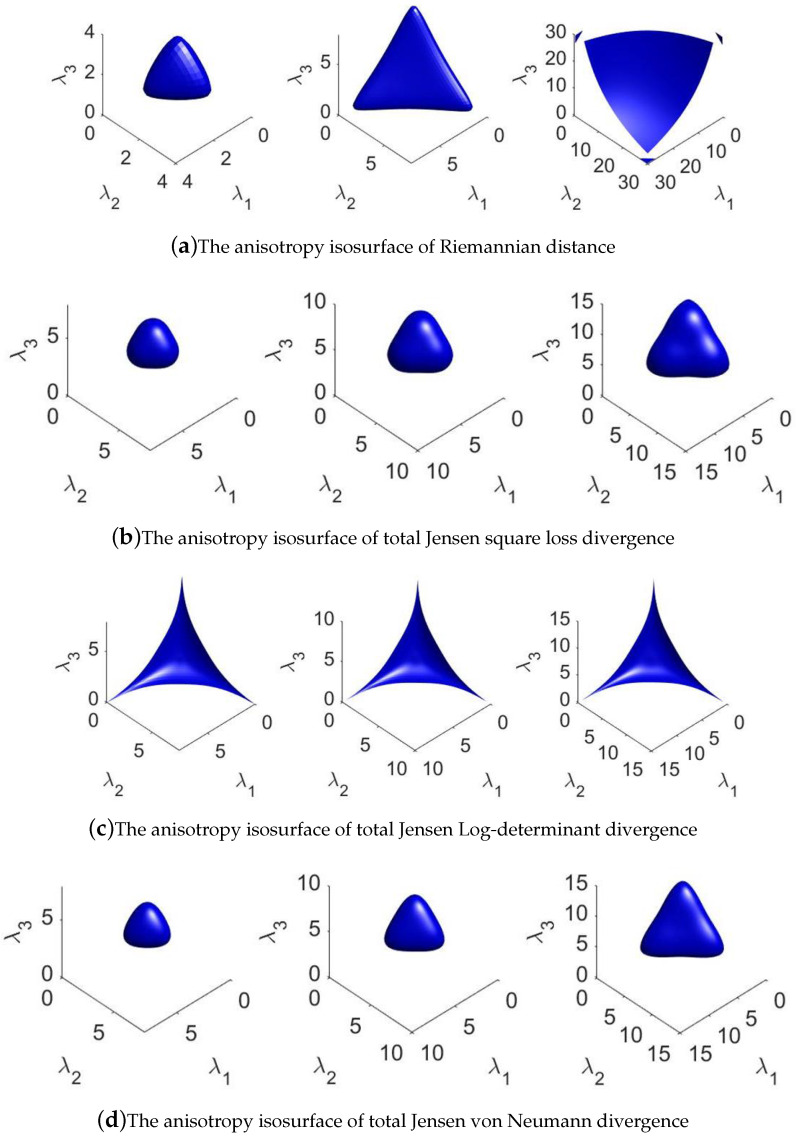
The isosurfaces of total Jensen-Bregman divergence.

In our previous work [18,19], anisotropies for six geometric measures on the Riemannian manifold are explored. An interesting phenomenon has been found that the detection performance may have some relationships with the anisotropy of a measure. However, the relationship needs further research. As a fact, the anisotropy implies the dissimilarity between metric tensors along different directions on the Riemannian manifold. It is well known that a linear space spanned by basis vectors is isotropic. However, the Riemannian manifold is a non-linear space, where different locations may have different anisotropies. In order to illustrate the difference of anisotropy of total Jensen–Bregman divergence, we give some visual results with 3-dimensional HPD matrix. Anisotropy isosurfaces, centered at the identity matrix with radius *r* = 1, 2 and 5 respectively, are shown in Figure 2.

It is clear from Figure 2 that isosurfaces of these measures are different from each other. Different measures have different anisotropies at the point *I* on the manifold P(3). This also means that the curvatures along the different directions are not identical. In other word, the metric matrix on the Riemannian manifold is not an identity matrix. The curvature can reflect the structure of manifold.

## 4. Total Jensen–Bregman Divergence Median on the Riemannian Manifold of HPD Matrices

Many literatures have been reported about the median matrix of HPD matrices. For instance, in [27], the Riemannian median is defined as the minimizer of the sum of geodesic distances to given matrices. In [9], Moakher defines the Log-Euclidean and Bhattacharyya medians for filtering the medical image. In the following, we give the definition of total Jensen–Bregman divergence median.

**Definition** **2.** *Let a convex function f be differentiable, for m HPD matrices $\left\{R_{1},R_{2},\ldots ,R_{m}\right\}$, the median associated with total Jensen-Bregman divergence (10) is defined as,*

(14) $$\begin{array}{c} \hat{R}=\underset{R}{\arg\min}\;\sum_{i=1}^{m}\delta_{\alpha }\left(R,R_{i}\right) \end{array}$$

Note that the right-sided centroids ${\hat{R}}^{\prime }$ are obtained by minimizing the equivalent left-sided centroids for $\alpha^{\prime }=1-\alpha$, and ${{\hat{R}}^{\prime }}_{\alpha }={\hat{R}}_{1-\alpha }$. Therefore, we consider the left-sided centroids in the remainder.

In order to minimize Equation (14), we proceed iteratively in two stages:

Stage 1: we consider ${\hat{R}}^{\left(t\right)}$ given, here, ${\hat{R}}^{\left(0\right)}=\frac{1}{m}\sum_{i=1}^{m}R_{i}$. This allows us to consider the following simpler minimization problem:

(15) $$\begin{array}{c} \hat{R}=\arg\min_{R\in \left(n\right)}\;\sum_{i=1}^{m}\;\;\;\{\;\;\;\rho \left({\hat{R}}^{\left(t\right)},R_{i}\right)\frac{1}{\alpha \left(1-\alpha \right)}\times tr\left({\left(f\left(R\right)f\left(R_{i}\right)\right)}_{\alpha }-f\left({\left(RR_{i}\right)}_{\alpha }\right)\right)\;\;\;\}\;\;\; \end{array}$$

Let

(16) $$\begin{array}{c} w_{i}^{\left(t\right)}=\frac{\rho \left({\hat{R}}^{\left(t\right)},R_{i}\right)}{\sum_{j}\rho \left({\hat{R}}^{\left(t\right)},R_{j}\right)} \end{array}$$

be the updated renormalized weights at stage *t*.

Stage 2: we minimize

(17) $$\begin{array}{c} \hat{R}=\arg\min_{R\in \left(n\right)}\;\sum_{i=1}^{m}w_{i}^{\left(t\right)}\frac{1}{\alpha \left(1-\alpha \right)}tr\left({\left(f\left(R\right)f\left(R_{i}\right)\right)}_{\alpha }-f\left({\left(RR_{i}\right)}_{\alpha }\right)\right) \end{array}$$

This is a convex-concave minimization procedure [28] (CCCP) that can be solved iteratively until it reaches convergence [29]. The above two iterations can be formulated as following:

(18) $$\begin{array}{c} {\hat{R}}^{\left(t+1\right)}={\left(\nabla f\right)}^{-1}\left(\sum_{i=1}^{m}w_{i}^{\left(t\right)}\nabla f\left(\alpha {\hat{R}}^{\left(t\right)}+\left(1-\alpha \right)R_{i}\right)\right) \end{array}$$

It is an open and challenging problem to prove that the total Jensen–Bregman medians are unique whatever the chosen multivariate *f*, see [29].

In the following, we will give total Jensen–Bregman medians according to different forms of *f*.

**Proposition** **1.** *For m HPD matrices $\left\{R_{1},R_{2},\ldots ,R_{m}\right\}$, the median associated with total Jensen square loss (11) is given by,*

(19) $$\begin{array}{c} {\hat{R}}^{\left(t+1\right)}=\sum_{i=1}^{m}w_{i}^{\left(t\right)}\left(\alpha {\hat{R}}^{\left(t\right)}+\left(1-\alpha \right)R_{i}\right),w_{i}^{\left(t\right)}=\frac{\rho \left({\hat{R}}^{\left(t\right)},R_{i}\right)}{\sum_{j}\rho \left({\hat{R}}^{\left(t\right)},R_{j}\right)}\\ \rho \left({\hat{R}}^{\left(t\right)},R_{i}\right)=1/\sqrt{1+\frac{{∥{\left({\hat{R}}^{\left(t\right)}\right)}^{2}-R_{i}^{2}∥}_{F}^{2}}{{∥{\hat{R}}^{\left(t\right)}-R_{i}∥}_{F}^{2}}}\; \end{array}$$

**Proof** **of** **Proposition** **1.** According to Equation (11), $f\left(R\right)=R^{2}$, then we have,

(20) $$\begin{array}{c} \nabla f\left(R\right)=2R,\nabla f^{-1}\left(R\right)=R/2\; \end{array}$$

 ☐

Substitution Equation (20) into Equation (18), and we can obtain the total Jensen square loss median as Equation (19).

**Proposition** **2.** *For m HPD matrices $\left\{R_{1},R_{2},\ldots ,R_{m}\right\}$, the median associated with total Jensen von Neumann divergence (12) is given by,*

(21) $$\begin{array}{c} {\hat{R}}^{\left(t+1\right)}=\exp\left(\sum_{i=1}^{m}w_{i}^{\left(t\right)}\log\left(\alpha {\hat{R}}^{\left(t\right)}+\left(1-\alpha \right)R_{i}\right)\right),w_{i}^{\left(t\right)}=\frac{\rho \left({\hat{R}}^{\left(t\right)},R_{i}\right)}{\sum_{j}\rho \left({\hat{R}}^{\left(t\right)},R_{j}\right)},\\ \rho \left({\hat{R}}^{\left(t\right)},R_{i}\right)=1/\sqrt{1+\frac{{∥\left({\hat{R}}^{\left(t\right)}\log\left({\hat{R}}^{\left(t\right)}\right)-{\hat{R}}^{\left(t\right)}\right)-\left(R_{i}\log\left(R_{i}\right)-R_{i}\right)∥}_{F}^{2}}{{∥{\hat{R}}^{\left(t\right)}-R_{i}∥}_{F}^{2}}}\; \end{array}$$

**Proposition** **3.** *The definition of total Jensen von Neumann divergence is given by choosing the convex function $f\left(R\right)=R\log\left(R\right)-R$. The first derivative of the function f and its inverse function are given as,*

(22) $$\begin{array}{c} \nabla f\left(R\right)=\log\left(R\right),\nabla f^{-1}\left(R\right)=exp\left(R\right) \end{array}$$

Substitution Equation (22) into Equation (18), and we have the median as Equation (21).

**Proposition** **4.** *The median associated with total Jensen log-determinant divergence (13), of m HPD matrices $\left\{R_{1},R_{2},\ldots ,R_{m}\right\}$ is given by,*

(23) $$\begin{array}{c} {\hat{R}}^{\left(t+1\right)}={\left(\sum_{i=1}^{m}w_{i}^{\left(t\right)}{\left(\alpha {\hat{R}}^{\left(t\right)}+\left(1-\alpha \right)R_{i}\right)}^{-1}\right)}^{-1},w_{i}^{\left(t\right)}=\frac{\rho \left({\hat{R}}^{\left(t\right)},R_{i}\right)}{\sum_{j}\rho \left({\hat{R}}^{\left(t\right)},R_{j}\right)}\\ \rho \left({\hat{R}}^{\left(t\right)},R_{i}\right)=1/\sqrt{1+\frac{{∥\log\left({\hat{R}}^{\left(t\right)}\right)-\log\left(R_{i}\right)∥}_{F}^{2}}{{∥{\hat{R}}^{\left(t\right)}-R_{i}∥}_{F}^{2}}}\; \end{array}$$

**Proof** **of** **Proposition** **3.** The total Jensen von Neumann divergence is obtained by choosing the convex function $f\left(R\right)=-\log\left(R\right)$, and then we have,

(24) $$\begin{array}{c} \nabla f\left(R\right)=R^{-1},\nabla f^{-1}\left(R\right)=R^{-1} \end{array}$$

 ☐

Substitution Equation (24) into Equation (18), and we have the median as Equation (23).

**Figure 3:**
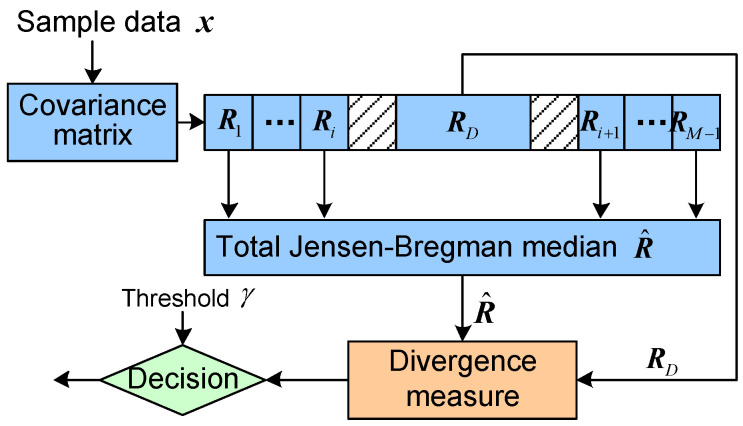
Total Jensen-Bregman divergence-based geometric detector.

According to the definition of total Jensen–Bregman divergence, we can design the corresponding geometric detector illustrated in Figure 3. The HPD matrix $R_{i}$ of each cell is estimated by the sample data x according to its correlation coefficient. The detection statistic is the divergence $\delta_{f}\left(R_{D},\hat{R}\right)$ of the matrix $R_{D}$ of cell under test and its median matrix $\hat{R}$. Thus, the statistic is compared with a given threshold γ for making the decision. The formulation of target detection can be given as,

(25) $$\begin{array}{c} \delta_{f}\left(R_{D},\hat{R}\right)\underset{targetabsent}{\overset{targetpresent}{≷}}\;\gamma \end{array}$$

## 5. Experimental Results

In this section, we analyze the performances of our proposed detection algorithms in terms of probability of detection ($P_{d}$). We also compare detection performances of our proposed geometric detectors, named as TJSL (total Jensen square loss), TJLD (total Jensen log-determinant divergence), and TJVN (total Jensen von Neumann divergence), in comparison with the classical FFT-CFAR detector [1] and the Riemannian distance-based target detection algorithm [2], abbreviated as the Riemannian detector.

### 5.1. Numerical Simulation

In simulation experiments, we make use of the Monte Carlo counting technique to test performances of the TJSL detector, the TJVN detector, the TJLD detector, the Riemannian detector, and the FFT-CFAR detector. We consider a radar system, the pulse repetition frequency (PRF) is 1000 Hz, and the radar central frequency $f_{c}$ is 9 GHz. Seven pulses data are contained in the received echo, and the target model is given as $\tilde{a}p$, where $\tilde{a}$ is a parameter relating to the backscattering and the channel propagation effects. *p* denotes the target steering vector,

(26) $$\begin{array}{c} p=\frac{1}{\sqrt{N}}{\left[1,\exp\left(j2\pi f_{d}T_{r}\right),\ldots ,\exp\left(j2\pi \left(N-1\right)f_{d}T\right)\right]}^{T} \end{array}$$

where $f_{d}$ denotes the Doppler frequency, and $T_{r}$ is the pulse repetition interval. We assume the target has a velocity v=5 m/s. The number of reference cells is considered for 16. In many high resolution radar scenarios, the Gaussian distribution assumption is not consistent with the actual situation, and cannot be used as clutter model. The compound Gaussian model is more suitable for describing non-Gaussian clutter. The compound Gaussian clutter can be written as the product of two independent random variables. The speckle component is a zero mean Gaussian process, and the texture component is a non-negative random process. It describes the average power level of clutter [30]. In our experiments, the non-Gaussian clutter is simulated via the *K* distribution, the probability density function of *K* distribution is given [31],

(27) $$\begin{array}{c} p\left(x\right)=\frac{\sqrt{2\nu /a\;}}{2^{\nu -1}\Gamma \left(\nu \right)}{\left(\sqrt{\frac{2\nu }{a}}x\right)}^{\nu }K_{\nu -1}\left(\sqrt{\frac{2\nu }{a}}x\right),x\geq 0 \end{array}$$

where $K_{\nu -1}\left(\cdot \right)$ is the modified Bessel function of the second kind with order v−1, and Γ(·) is the gamma function. *v* is the shape parameter, and *a* denotes the scale parameter.

Simulation experiments are performed to validate detection performances of the TJSL detector, the TJVN detector, and the TJLD detector via the Monte Carlo technique. Results are compared with the Riemannian and FFT-CFAR detectors. As the threshold cannot be analyzed in theory, several $10^{6}$-trial Monte Carlo simulations in the absence of a target are used to obtain the threshold for a given false alarm probability ($P_{fa}$). To estimate the detection probability accurately, the $P_{d}$ is computed by the relative frequency using 200 runs of Monte Carlo simulations.

**Figure 4:**
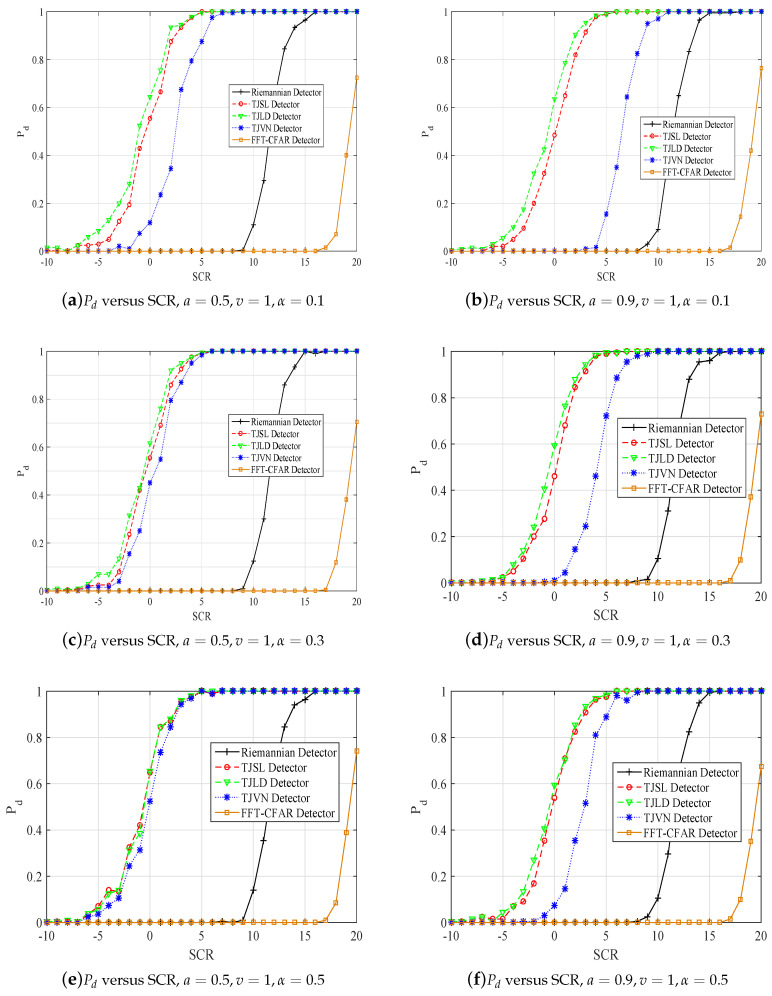
$P_{d}$ versus SCR in K distribution, $P_{fa}=10^{-5}$.

Figure 4 gives plots of $P_{d}$ vs SCRs for $P_{fa}=10^{-5}$. The SCR varies from −20 to 10 dB. Inspection of Figure 4 highlights that our proposed detectors outperform the Riemannian detector. Particularly, the TJLD detector has the best performance when α=0.1,a=0.5,v=1; α=0.1,a=0.9,v=1; and α=0.3,a=0.5,v=1; α=0.3,a=0.9,v=1 and followed by the TJSL detector. In addition, the rest of the presented results show that the TJLD detector has similar performance with the TJSL detector.

The strategy of our proposed geometric detector in this paper is that the decision about “target absence” or “target presence” is made according to the dissimilarity between the matrix of cell under test and the clutter covariance matrix estimated by its surrounding cells. The dissimilarity is very different with different used metrics. In our previous work [18,19], we have explored the relationship between the anisotropy of a measure and the performance of its corresponding geometric detector. The estimation accuracy of the covariance matrix is not the factor that affects the performance of the geometric detector, but the anisotropy, which reflects the geometric structure of manifold. Further work will be reported in our other papers.

In the following, we will illustrate the robustness of covariance estimation with respect to the number of samples collected. Given a covariance matrix $R_{0}$, several samples are generated by an N-dimension zero-mean Gaussian distribution with covariance matrix $R_{0}$. Then, we estimate the covariance matrix $\hat{R}$ according to these samples. Finally, the estimation accuracy denoted by the error value is computed as,

(28) $$\begin{array}{c} error=\frac{{∥R_{0}-\hat{R}∥}_{F}}{{∥R_{0}∥}_{F}} \end{array}$$

**Figure 5:**
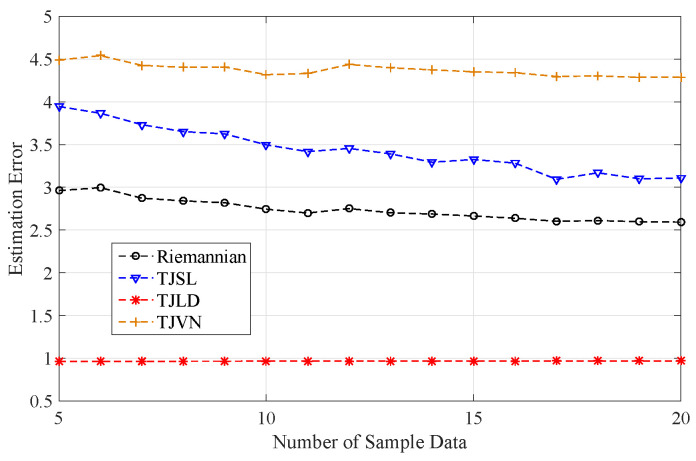
The estimation accuracy of the covariance matrix under different number of samples.

The number of samples varies from five to 20, and the number of pulses N is seven. A total of 100 times simulations are repeated to compute the average of the error value. Figure 5 gives the plot of the estimation accuracy of the covariance matrix under different number of samples.

It is clear from Figure 5 that the variance of estimation error is not obvious, as the number of samples varies. This means that the estimated matrix, that is the Riemannian mean, the TJSL median, the TJLD median, and the TJVN median, is robust with respect to the number of samples, especially the TJLD median. Moreover, it can be observed from Figure 5 that the TJLD median has the lowest estimation error, followed by the Riemannian mean. The TJVN median has the maximal estimation error. However, the relationship of estimation error is not consistent with the detection performance which is illustrated in Figure 4.

### 5.2. Real Clutter Data

To evaluate the performance of the proposed detectors in a real application situation, we use data collected by the McMaster University IPIX radar [32]. Specifically, 19980205-192053-antstep.cdf (file 1), and 19980205-185111-antstep.cdf (file 2) [33] are employed to test detection performances of our proposed detectors, Riemannian detector, and FFT-CFAR detection algorithm. For these two data files, the number of samples in the range dimension is 27, and the pulse dimension consists of 60,000 samples. The pulse repetition frequency is 1000 Hz, and the central frequency is 9.39 GHz. The horizontal polarization (HH) information of samples are considered. In particular, a synthetic target is needed to inject into one cell due to the unavailable target information, and the velocity is approximate constant, v=5 m/s.

The dimension of the HPD matrix is 5, the threshold is set using the first 50,000 pulse in each cell for $P_{fa}=10^{-3}$. Other parameters are also as in the former subsection. The simulation is repeated 200 times to determine the Pd by means of the relative frequency.

**Figure 6:**
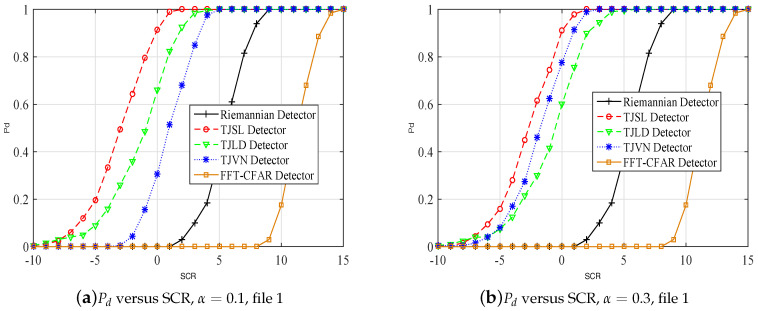

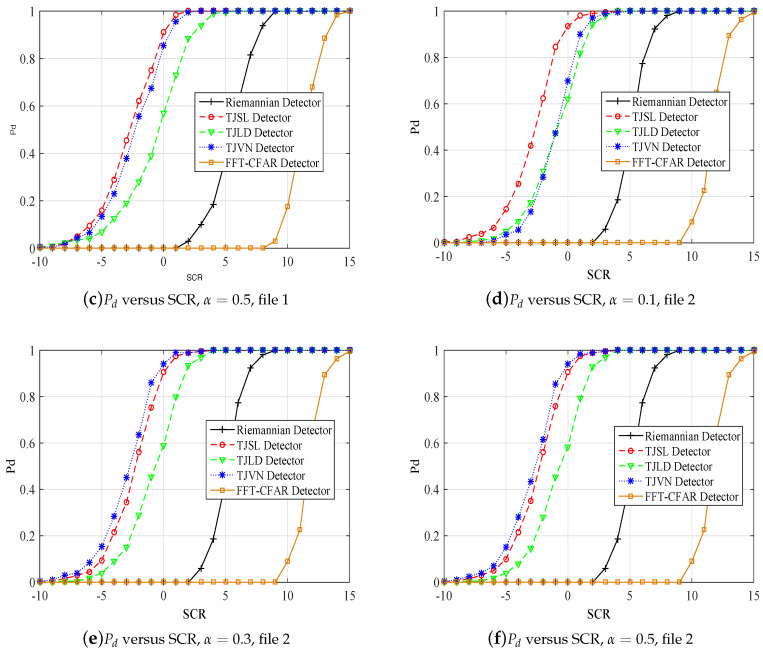
$P_{d}$ versus SCR in real clutter environment, $P_{fa}=10^{-3}$.

We compare the performance of our proposed detectors using the above two real sea clutter data. Plots of detection probability for different detectors are shown in Figure 6. The $P_{fa}$ is set to $10^{-3}$, and the range of SCR is between −10 and 15 dB. From Figure 6, it is clear that our proposed detector and the Riemannian detector outperform the classical FFT-CFAR detection algorithm in real clutter environment. The detection performance of TJSL detector is the best in file1, and the performance of TJSL detector is comparable with the TJVN detector with α=0.5. Moreover, the results imply that our proposed detectors may have different performances in different real data files. The performance analysis of our proposed detectors under different clutter environments is needed in further work.

## 6. Conclusions

In this paper, we have developed a radar target detection algorithm using the matrix information geometry method. The total Jensen–Bregman divergence is defined and used as the distance-like function on the Riemannian manifold of Hermitian positive-definite matrix. Definitions of three divergences, including the TJSL, the TJLD, the TJVN, are given according to different forms of the convex function *f*. Then, medians associated with the total Jensen–Bregman divergence are derived. For the stage of the performance analysis, we compare detection performances of our proposed detectors with the Riemannian detector and the FFT-CFAR detection algorithm using the simulation data and the real sea clutter data file. The comparison results have shown a remarkable advantage with respect to the conventional FFT-CFAR detection algorithm and the Riemanian detector.

## References

[1] Richards M. (2005). Fundamentals of Radar Signal Processing.

[2] Lapuyade-Lahorgue J., Barbaresco F. Radar detection using Siegel distance between autoregressive processes, application to HF and X-band radar. Proceedings of the 2008 RADAR ’08 IEEE Radar Conference.

[3] Barbaresco F. New Foundation of radar Doppler signal processing based on advanced differential geometry of symmetric spaces: Doppler matrix CFAR and radar application. Proceedings of the 2009 RADAR ’09 IEEE Radar Conference.

[4] Arnaudon M., Barbaresco F., Yang L. (2011). Medians and Means in Riemannian Geometry: Existence, Uniqueness and Computation. Matrix Inf. Geom..

[5] Liu Z., Barbaresco F. (2013). Doppler Information Geometry for Wake Turbulence Monitoring.

[6] Barbaresco F. (2012). Method for Radar Monitoring of Wake Turbulence. U.S. Patent.

[7] Barbaresco F., Meier U. (2010). Radar monitoring of a wake vortex: Electromagnetic reflection of wake turbulence in clear air. Comptes Rendus Phys..

[8] Charfi M., Chebbi Z., Moakher M., Vemuri B.C. Using the Bhattacharyya Mean for the Filtering and Clustering of Positive-Definite Matrices. Proceedings of the GSI 2013 Geometric Science of Information: First International Conference.

[9] Charfi M., Chebbi Z., Moakher M., Vemuri B.C. Bhattacharyya median of symmetric positive-definite matrices and application to the denoising of diffusion-tensor fields. Proceedings of the IEEE International Symposium on Biomedical Imaging.

[10] Kullback S., Leibler R.A. (1951). On Information and Sufficiency. Ann. Math. Stat..

[11] Vemuri B.C., Liu M., Amari S.I., Nielsen F. (2011). Total Bregman Divergence and Its Applications to DTI Analysis. IEEE Trans. Med. Imaging.

[12] Teran A.R.M.Y., Gouiffes M., Lacassagne L. Total Bregman Divergence for Multiple Object Tracking. Proceedings of the International Conference on Image Processing.

[13] Liu M., Vemuri B.C., Amari S.I., Nielsen F. Total Bregman divergence and its applications to shape retrieval. Proceedings of the 2010 IEEE Conference on Computer Vision and Pattern Recognition.

[14] Liu M., Vemuri B.C., Amari S.I., Nielsen F. (2012). Shape Retrieval Using Hierarchical Total Bregman Soft Clustering. IEEE Trans. Pattern Anal. Mach. Intell..

[15] Nielsen F., Nock R. Total Jensen divergences: Definition, properties and clustering. Proceedings of the IEEE International Conference on Acoustics, Speech and Signal Processing.

[16] Nielsen F., Nock R. (2013). Total Jensen divergences: Definition, Properties and k-Means++ Clustering. arXiv.

[17] Cheng Y., Hua X., Wang H., Qin Y., Li X. (2016). The Geometry of Signal Detection with Applications to Radar Signal Processing. Entropy.

[18] Hua X., Cheng Y., Wang H., Qin Y., Li Y. (2017). Geometric means and medians with applications to target detection. IET Signal Process..

[19] Hua X., Cheng Y., Wang H., Qin Y., Li Y., Zhang W. (2017). Matrix CFAR detectors based on symmetrized Kullback Leibler and total Kullback Leibler divergences. Digit. Signal Process..

[20] Hua X., Cheng Y., Wang H., Qin Y., Chen D. (2018). Geometric target detection based on total Bregman divergence. Digit. Signal Process..

[21] Bhatia R. (2007). Positive Definite Matrices.

[22] Sra S. (2011). Positive definite matrices and the Symmetric Stein Divergence. arXiv.

[23] Bridson M.R., Haefliger A. (1999). Metric Spaces of Non-Positive Curvature.

[24] Terras A. (2014). Harmonic Analysis on Symmetric Spaces and Applications II.

[25] Wolkowicz H., Saigal R., Vandenberghe L. (2000). Handbook of Semidefinite Programming: Theory, Algorithms and Applications.

[26] Lang S. (1999). Fundamentals of Differential Geometry.

[27] Fletcher P.T., Venkatasubramanian S., Joshi S. Robust statistics on Riemannian manifolds via the geometric median. Proceedings of the IEEE Conference on Computer Vision and Pattern Recognition.

[28] Yuille A.L., Rangarajan A. (2003). The Concave-Convex Procedure. Neural Comput..

[29] Nielsen F., Boltz S. (2011). The Burbea-Rao and Bhattacharyya Centroids. IEEE Trans. Inf. Theory.

[30] Roy L.P., Raja Kumar R.V. A GLRT detector in partially correlated texture based compound-Gaussian clutter. Proceedings of the 2010 National Conference on, Communications (NCC).

[31] Watts S. (1987). Radar Detection Prediction in K-Distributed Sea Clutter and Thermal Noise. IEEE Trans. Aerosp. Electron. Syst..

[32] Haykin S., Krasnor C., Nohara T.J., Currie B.W. (1991). A coherent dual-polarized radar for studying the ocean environment. IEEE Trans. Geosci. Remote Sens..

[33] (1993). IPIX Radar File. IPIX Radar Dataset Files in Dartmouth. http://www.ipixbpo.com/.

